# Supplementary material for: A nonparametric significance test for sampled networks
Source: Bioinformatics. 2017 Jul 7;34(1):64–71. doi: 10.1093/bioinformatics/btx419 (PMC5870844; doi:10.1093/bioinformatics/btx419)
Supplement: Supplementary Data [file samplingnullmodelsuppinfo_btx419.pdf]

Supplementary Information: A Nonparametric Significance Test for Sampled Networks

Andrew Elliott[1,*], Elizabeth Leicht[1], Alan Whitmore[4], Gesine Reinert[2], and Felix Reed-Tsochas[1,3]

[1]CABDyN Complexity Centre, Saïd Business School, University of Oxford
[2]Department of Statistics, University of Oxford
[3]Oxford Martin School, University of Oxford
[4]e-Therapeutics plc, 17 Blenheim Office Park, Long Hanborough, OX29 8LN
[*]To whom correspondence should be addressed.

July 5, 2017

1 Seed List Construction

This section contains the tables with the seed lists and the conversions into BioGRID identifiers. Note, some of the identifiers convert to more than one node in BioGRID, and some are not present.

The seeds for OMIM can be found in Table 1. In the case of the table for the expression seed list as this article is open access, copyright issues prevent us from reprinting the original table from Ref. [2], thus we include the conversions we made from the table into gene names and from these gene names to BioGRID IDs (Table 2) and we invite the reader to observe Table 1 in Ref. [2].

Note that when constructing this list we were unable successfully to map two of the genes in the expression seed into gene names. Subsequently we have been able to do so and this will be incorporated into future work. However, the results in this document are based on the list without these genes, as is reflected in the table.

OMIM Entry	Mapped BioGrid Ids
Parkinson disease 1, 168601 (3)—SNCA, NACP, PARK1, PARK4—163890—4q22.1	112506
Parkinson disease 10 (2)—PARK10, AAOPD—606852—1p32	NONE
Parkinson disease 11, 607688 (3)—GIGYF2, KIAA0642, PARK11—612003—2q37.1	117520
Parkinson disease 12 (2)—PARK12—300557—Xq21-q25	NONE
Parkinson disease 13, 610297 (3)—HTRA2, OMI, PARK13, PRSS25—606441—2p13.1	118165
Parkinson disease 14, 612953 (3)—PLA2G6, IPLA2, INAD1, NBIA2B, NBIA2A, PARK14—603604—22q13.1	113986*
Parkinson disease 15, autosomal recessive, 260300 (3)—FBXO7, FBX7, FBX, PKPS, PARK15—605648—22q12.3	117326
Parkinson disease 17, 614203 (3)—VPS35, MEM3, PARK17—601501—16q11.2	120855
Parkinson disease 18, 614251 (3)—EIF4G1, EIF4G, PARK18—600495—3q27.1	108296
Parkinson disease 3 (2)—PARK3—602404—2p13	NONE
Parkinson disease 4, 605543 (3)—SNCA, NACP, PARK1, PARK4—163890—4q22.1	112506
Parkinson disease 6, early onset, 605909 (3)—PINK1, PARK6—608309—1p36.12	122376
Parkinson disease 7, autosomal recessive early-onset, 606324 (3)—DJ1, PARK7—602533—1p36.23	116446
Parkinson disease 8, 607060 (3)—LRRK2, PARK8—609007—12q12	125700
Parkinson disease 9, 606693 (3)—ATP13A2, PARK9, KRPPD—610513—1p36.13	116973
Parkinson disease, juvenile, type 2, 600116 (3)—PRKN, PARK2, PDJ, LPRS2—602544—6q26	111105
Parkinson disease 16 (2)—PARK16—613164—1q32	NONE
Parkinson disease 5, susceptibility to, 613643 (3)—UCHL1, PARK5—191342—4p13	113192
Parkinson disease, late-onset, susceptibility to, 168600 (3)—GBA—606463—1q22	108899*
Parkinson disease, susceptibility to, 168600 (3)—ADH1C, ADH3—103730—4q23	NONE
Parkinson disease, susceptibility to, 168600 (3)—MAPT, MTBT1, DDPAC, MSTD—157140—17q21.31	110308*
Parkinson disease, susceptibility to, 168600 (3)—TBP, SCA17, HDL4—600075—6q27	112771

Table 1: OMIM Seed List including mappings to BioGRID. OMIM records are taken from the OMIM database download [5] (full citation is as follows: Online Mendelian Inheritance in Man, OMIM. McKusick-Nathans Institute of Genetic Medicine, Johns Hopkins University (Baltimore, MD), 2012. World Wide Web URL: https://omim.org/) Conversion is performed using the mapping present in the BioGRID database. Proteins marked with a * are present in BioGrid but are not part of the largest connected component of the network composed of interactions from Yeast 2 Hybrid.

No.	Gene Name	Mapped BioGrid Ids
1	LGALS3	110149
2	SOX4	112542
3	TWIST1	113142
4	PGK1	111251
5	NOT IDENTIFIED	NONE
6	SNRPB2	112513
7	CLK3	107609
8	CDKN1A	107460, 111099
9	NFE2L1	110851
10	ASNS	106932
11	ID3	109625
12	ID1	109623
13	PTMA	111724, 111728*
14	IGFBP5	109709
15	JUN	109928
16	MYC	110694
17	RBM14	115700
18	NR2F1	112883, 112884
19	HSPA1A	109535, 109536
20	HSPA9	109545
21	SLC12A4	112449*
22	COMT	107707
23	PLOD1	111366
24	GOT1	109067, 119232*
25	MTHFD2	116011*
26	CCT5	116603
27	RPN2	112100
28	GADD45A	108014
29	GSTT2	NONE
30	DDIT3	108016
31	SIAH2	112373
32	SSRP1	112627
33	IL2RA	109774*
34	DLK1	114316
35	GPI	115325, 109082*
36	VEGFA	113265
37	RNH1	111977*, 128882, 129787*
38	NOT IDENTIFIED	NONE
39	PRKCA	111564
40	PPP2R2B	111513
41	RAC1	111817
42	YWHAB	113361
43	CTTN	108332
44	HINT1	109341
45	SOCS2	114362
46	CRYM	107815
47	SOCS7	119053, 125796
48	TSC2	113100

Table 2: Table contains our conversions of the genes in Table 1 from Ref [2] into gene names and the subsequent conversion into a BioGRID identifier using the BioGRIDs internal conversions. The ordering is the same in each table. Proteins marked with a * are present in BioGRID but are not part of the largest connected component of the network composed of interactions from Yeast 2 Hybrid.

2 Derivation Of Analytic Results

The analytic results shown here have been extended from results in Ref. [4] and follow some of the notation. Note, Ref. [4] focuses on the classic problem of an unknown network with observed samples, in this case we have the related problem where we know that the network but we want to explore features of the samples. We define the function $h(J, s)$ as the probability that there does not exist a node within n hops of J on a randomly selected seed list of size s. Let $B_n(J)$ be the set of nodes within n hops of the nodes in J. If

the selection is uniformly at random from all subsets of nodes of size s then we can use a hypergeometric distribution to derive an expression for $h(J,s)$. We can do so as follows, we randomly select s seeds from $|V|$, however if we select a seed from $B_n(J)$ then at least one node in J will be included. Thus we require the seeds to all be chosen from $V \setminus B_n(J)$. Thus through the hypergeometric distribution this results in the following form for $h(J,s)$:

$$h(J,s) = \frac{\binom{|V|-|B_n(J)|}{s}\binom{|V|-(|V|-|B_n(J)|)}{s-s}}{\binom{|V|}{s}},$$

which simplifies to:

$$h(J,s) = \frac{(|V|-s)!(|V|-|B_n(J)|)!}{(|V|-|B_n(J)|-s)!|V|!} = \prod_{i=0}^{s-1} \frac{|V|-|B_n(J)|-i}{|V|-i},$$

If we wish to fix the degree sequence of the seeds (or with a small adjustment binned degree), we can use a similar approach to the uniform case, to derive an expression for a degree sequence version, $h_d(J,t)$ where t is the degree sequence. This results in the following form for $h(J,t)$:

$$h_d(J,t) = \prod_{u\in U(t)} \prod_{i=0}^{F(t,u)-1} \frac{D(V,u)-D(B_n(J),u)-i}{D(V,u)-i},$$

where t is the degree sequence of the seed list. Here $F(t,u)$ is a counting function, it counts how many instances of u there are in t (e.g. $F([1,2,3,4,1],1)=2$), $U(t)$ returns the unique elements of l and $D(J,r)$ is the number of elements in J of degree r. The seeds of different degrees are selected independently so we can calculate the probability for each unique degree in the seed degree list and then multiply them to get the final probability.

Deriving the Mean and Variance Following the notation in the paper we let X be a random variable denoting the number of nodes in a snowball sampled graph with seed list S, where S is a uniform random draw over all possible seed lists. For notational convenience we define $|S|$ as the number of seeds in S. We are interested in the case where $|S|=s$, thus we will constrain our calculations to this case. Note, by further restricting S we obtain other schemes for example enforcing the degree sequence. In the case of enforcing the degree sequence we can repeat many of the following arguments by replacing h with h_d.

If we let Y_i be an indicator variable for the presence of node i in the sample, then the number of nodes in a sample is $X=\sum_i Y_i$. We can compute the mean number of nodes as follows:

$$E\big[X\big|\ |S|=s\big] = E\big[\sum_i Y_i\big|\ |S|=s\big] = \sum_i E\big[Y_i\big|\ |S|=s\big] = \sum_i 1-h(\{i\},s) = |V| - \sum_i h(\{i\},s).$$

To compute the variance we can use the correlated variables formula to get:

$$\mathrm{Var}(\sum_i Y_i\big|\ |S|=s) = \sum_i \mathrm{Var}(Y_i\big|\ |S|=s) + 2\sum_{i<j}\mathrm{Cov}(Y_i,Y_j\big|\ |S|=s).$$

We can imagine each term in the summation $(Y_i\big|\ |S|=s)$ as a Bernoulli random variable with $p=1-h(\{i\},s)$, therefore,

$$\mathrm{Var}(Y_i\big|\ |S|=s) = p(1-p) = h(\{i\},s)(1-h(\{i\},s)).$$

The covariance between the two variables is defined as:

$$\mathrm{Cov}(Y_i,Y_j\big|\ |S|=s) = E\big[Y_iY_j\big|\ |S|=s\big] - E\big[Y_i\big|\ |S|=s\big]E\big[Y_j\big|\ |S|=s\big];$$

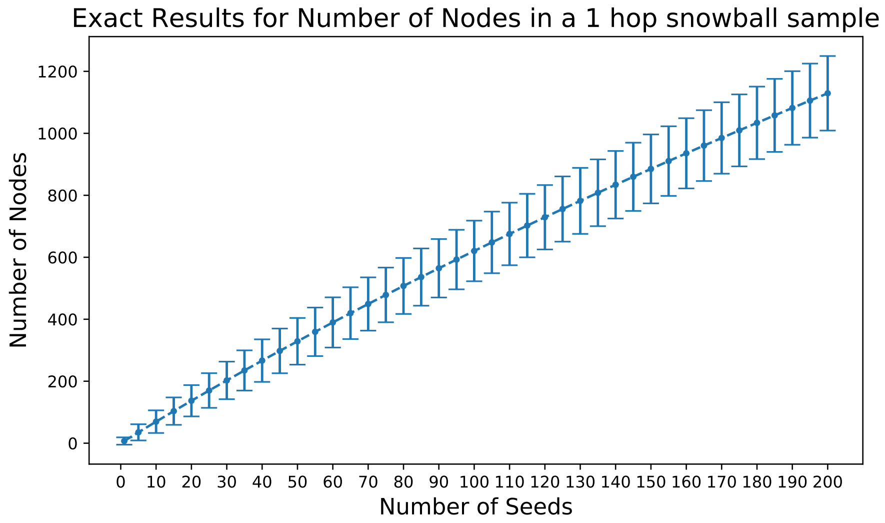

Figure 1: Exact Results: Points represent the mean number of nodes given a number of seed nodes in a 1-hop snowball sample from the BioGRID PPI network. The error bars represent one standard deviation of the same quantity.

thus:

$$\begin{aligned} E\big[Y_iY_j\big|\ |S|=s\big] &= E\big[(1-(1-Y_i))(1-(1-Y_j))\big|\ |S|=s\big] \\ &= 1 - E\big[1-Y_i\big|\ |S|=s\big] - E\big[1-Y_j\big|\ |S|=s\big] + E\big[(1-Y_i)(1-Y_j)\big|\ |S|=s\big] \\ &= 1 - h(\{i\},s) - h(\{j\},s) + h(\{i,j\},s). \end{aligned}$$

Combining and simplifying all of the terms we obtain:

$$\mathrm{Var}\Big(\sum_i Y_i\Big|\ |S|=s\Big) = (\sum_i h(\{i\},s) - h(\{i\},s)^2) + 2(\sum_{i<j} h(\{i,j\},s) - h(\{i\},s)h(\{j\},s)).$$

We can then factor the expression to obtain the form in the paper:

$$\mathrm{Var}\Big(\sum_i Y_i\Big|\ |S|=s\Big) = \sum_{\alpha=1}^{2} \alpha \sum_{\substack{J\subseteq V \\ |J|=\alpha}} h(J,s) - (\sum_i h(\{i\},s))^2.$$

where L is a dummy summation variable.

Figure 1 illustrates the effect of seed list size on the distribution of the number of nodes in a 1-hop snowball sample in the BioGRID PPI network [1, 7]. As we do not have a seed list of interest in this case, we have assumed that there is no restriction on the degree sequence of the seed nodes.

As expected, the larger the number of seed nodes, the larger the average number of nodes in the resulting network. Further, a small change in the number of seed nodes can have a large impact on the expected size of the network. The number of nodes in a 1-hop snowball sample on, say 20 proteins from the PPI network may appear small when compared to subnetworks randomly generated from 30 seeds but large when compared to such subnetwork generated from 10 seeds.

3 Algorithms To Add Or Remove Redundant Seeds

To discover redundant seed nodes we need to be able to guarantee that a pair of labelled networks are identical. The trivial way to do this is to compare the node lists and the edge lists, and if they are equal

then the networks are equal. As we will have to check equality a large number of times, this approach can can prove computationally constraining. Making use of the fact that we are adding or removing nodes from the seed list, we can derive a simpler condition for this problem.

We take an arbitrary network with node set V and edge list E and a seed list l_1. Let $[V_{l_1}, E_{l_1}] = f_{arb}(V, E, l_1)$, where f_{arb} is a arbitrary network sampling function, l_1 is a seed list and V_{l_1} and E_{l_1} are the subset of nodes and edges that are in the subnetwork.

Let us assume that we have two seed lists l_1 and l_2 and $l_1 \subseteq l_2$. Further, let us assume that our sampling technique (f_{arb}) guarantees that $V_{l_1} \subseteq V_{l_2}$ and $E_{l_1} \subseteq E_{l_2}$ if $l_1 \subseteq l_2$.

Trivially for sets T_1 and T_2 if $T_1 \subseteq T_2$ and $|T_1| = |T_2|$, then $T_1 = T_2$. Thus, if $|E_{l_1}| = |E_{l_2}|$ and $|V_{l_1}| = |V_{l_2}|$ then $E_{l_1} = E_{l_2}$ and $V_{l_1} = V_{l_2}$. Therefore we can use the condition:

$$|E_{l_1}| = |E_{l_2}| \text{ and } |V_{l_1}| = |V_{l_2}|. \tag{1}$$

Note, we must condition both on the set of edges and the set of nodes as they are both required to fully define the subnetwork.

Therefore if we can guarantee using our sampling techniques that if $l_1 \subseteq l_2$ then $V_{l_1} \subseteq V_{l_2}$ and $E_{l_1} \subseteq E_{l_2}$ then we can simply test for equality in the number of nodes and edges.

Snowball Sampling In snowball sampling the contribution from each of the nodes on the seed list is independent, as it is simply the number of nodes within a certain radius of the each of the seeds. Therefore the expression for the V_{l_1} is as follows:

$$V_{l_1} = \bigcup_{x \in l_1} V_x.$$

If we take $l_1 \subseteq l_2$, then $V_{l_2} = V_{l_1} \cup V_{l_2 \setminus l_1}$ and therefore trivially $V_{l_1} \subseteq V_{l_2}$. We can use the same argument for E_{l_1}. We can therefore use the condition in (1) for snowball sampled networks given that the seed list is a subset or a superset of the original seed list.

Deterministic Path Based Sampling Techniques We define a deterministic path based sampling techniques in undirected networks as a procedure for which the sample as is function of every pair of nodes on the seed list and the underlying network. Note that Path≤k and shortest path sampling both fall into this category. Therefore,

$$V_{l_1} = \{x \in P^{(V)}_{arb}(y, z) : y, z \in l_1\}, \text{ and } E_{l_1} = \{x \in P^{(E)}_{arb}(y, z) : y, z \in l_1\},$$

where $P^{(V)}_{arb}(y, z)$ and $P^{(E)}_{arb}(y, z)$ are sets of the sampled nodes and edges respectively on the path(s) between y and z defined by the sampling technique in question. Let l_1 and l_2 be seed lists and let $l_1 \subseteq l_2$, then

$$V_{l_2} = V_{l_1} \cup V_{l_2 \setminus l_1} \cup \{x \in P^{(V)}_{arb}(y, z) : y \in l_1,\ z \in l_2 \setminus l_1\} \cup \{x \in P^{(V)}_{arb}(y, z) : z \in l_2,\ y \in l_2 \setminus l_1\}.$$

where the right hand side is the union of the nodes included by the seed list l_1 (first term), the nodes that are included by the seed list $l_2 \setminus l_1$ (second term), and the third and fourth terms represent the contributions from paths which connect a node from l_1 to a node from $l_2 \setminus l_1$. Therefore $V_{l_1} \subseteq V_{l_2}$, and by similar argument $E_{l_1} \subseteq E_{l_2}$. Thus we can use the condition in (1) for all deterministic path sampling techniques.

3.1 Algorithm To Add Additional Redundant Seed Nodes

In the paper given a seed list, a network, a sampling technique and the resultant subnetwork, we construct the largest seed list that will generate the same subnetwork. For a given seed list and network the procedure to do this is as follows:

1. Make an empty list, which will contain the possible seed nodes.

2. For each node in the subnetwork, compute if it can be added to the seed list without increasing the number of nodes or edges in the subnetwork. If so add to the list of possible seed nodes.

3. Set $W = 0$.

4. Form all seed lists with the original seeds and all but W of the nodes on the possible seed list.

5. Test if each of these seed lists produces the same subnetwork. If at least one seed list produces the subnetwork return all tested seed lists that produce the same subnetwork, else let $W = W + 1$ and go back to step 4.

For all results in the paper we used the algorithm above, however for some sampling techniques we can construct a simplified procedure. For n-hop snowball sampling, we can construct a list of possible seed nodes using the following procedure:

```
listofSeeds=[]
OutsideNodes=list of nodes in (n+1)-hop snowball sample but not in n-hop sample
for curNode in Subnetwork
    if a node in OutsideNodes is in the n-hop snowball sample of curNode:
        continue
    else:
        add curNode to listofSeeds
```

3.2 Optimising Finding Redundant Seed Nodes

The algorithm presented in Section 2.4 of the paper is the following:

1. Remove each seed in turn and check if the number of nodes and edges in the subnetwork do not change. If not, then add the node to the list of redundant seeds.

2. Form a list of the remaining seeds.

3. Define a dummy variable L and set $L = 0$

4. For lists of redundant seeds of length L
   (a) Test if sampling with the list of the remaining seeds and the selected redundant seeds produce the same network.
   (b) Store all seed lists which pass the test.

5. If there are no seed lists which pass the test, set $L \to L + 1$ and go to step 4.

6. Return the smallest seed list(s) that produce the same network.

The major problem in this procedure is the large number of options that may need to be checked to find the minimum seed list. As stated in the paper finding the minimum seed list for snowball sampled networks can be converted into the set cover problem which is NP-hard [3].

The set cover problem is defined as follows, for a set $\mathfrak{F}$, and a collection of subsets $F = \{F_1, ..., F_m\}$ such that $\mathfrak{F} = \bigcup_{x \in F} x$ [3]. The problem is then to find the smallest subset of F which we shall call F^* such that $\mathfrak{F} = \bigcup_{x \in F^*} x$ [3].

We can reformulate the minimum seed list for Snowball sampled networks as follows. We let F be an empty set. For each seed node we compute the set of nodes which are sampled by this seed node and we add it to F. Finding the minimum seed list is then equivalent to finding F^* and then returning the seeds which were used to construct each element of F^*.

Thus in cases where we require additional speed we could try reformulating this as a set cover problem and use state of the art algorithms for this problem.

It may also be possible to convert the path based techniques into another related NP Complete or NP Hard problem and use a similar technique. However, as we do not require the speed for the work we are doing here we have not attempted to do so.

Further Optimisations A further optimisation, which is sampling technique dependent, can be performed on sampling techniques that scale with number of nodes in the whole network and that only depend on the information in the subnetwork. Sampling in the subnetwork rather than in the wider network can be more efficient while still guaranteeing the result. One example of this procedure is shortest path sampling. All of the information about shortest paths is included in the subnetwork. Therefore sampling with the reduced seed list in the subnetwork saves time (as shortest path scales with number of nodes and edges depending on implementation) and guarantees that the result is correct as long as the seed list is a subset of the original seed list.

4 Further Results: Adding Redundant Seed Results

We showed that redundant seed nodes have to be taken into account; in particular in Figure 4 of the paper we demonstrated that the significance of randomly chosen seed lists can be changed in the BioGRID network under 2-hop snowball sampling by increasing the size of the seed list without changing the resultant sampled network. A similar effect can be observed several other sampling techniques as can be seen in Figure 2.

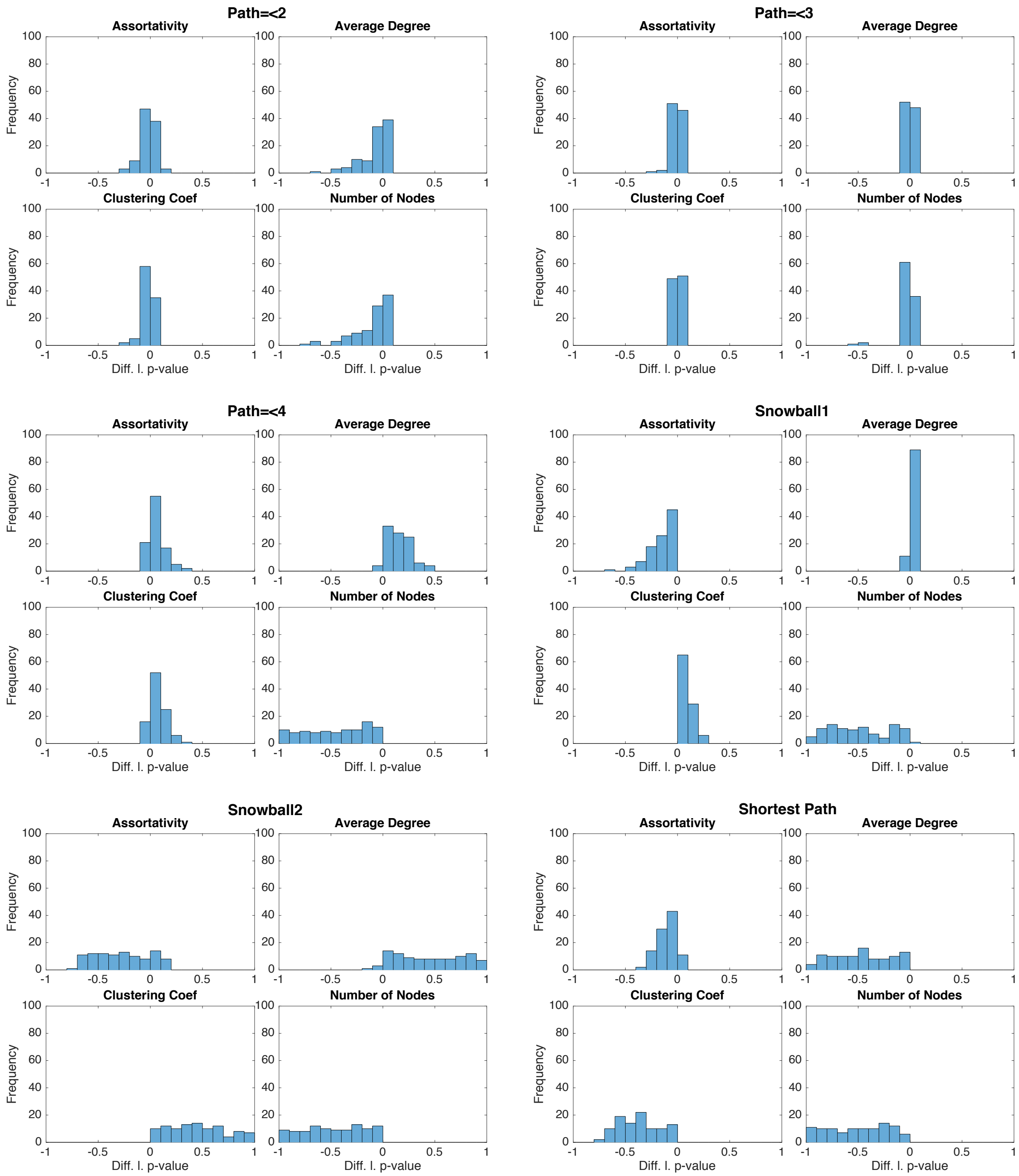

Figure 2: Histogram of differences in p-values of 100 of each of the sampling techniques in the BioGRID PPI network with 25 initial random seed proteins and a bin size of 20 generated by adding additional redundant seed nodes. Each of the p-values are computed using 2000 Monte Carlo realisations. In several cases we observe a large change in p-value using this procedure.

5 Empirical Seed Lists: Additional Results

To test whether the results we see in the paper are robust with respect to the bins that we use to generate the random seed lists, we recalculate the results in the paper using a minimum bin size of 5, 10, 20, 30 and 50. The smaller the bin size the closer the degree sequence will match the test sequence, whereas the larger minimum bin sizes produce seed lists which have degree sequences which are further from the test list, but have a lower likelihood of selecting the same small set of seed nodes. Figure 3 shows the results for the OMIM seed list and Figure 4 shows the results for the expression seed list.

6 Comparison With Configuration Model

The configuration model may not preserve the structural features of the original network. For example in the 2-hop snowball sample in Figure 5 there is a very clear structure, with a maximum path length of 4 between any pair of nodes. This structure will not be preserved in the configuration model Fig. 6 shows a simple comparison between the distribution of shortest path length in the snowball sampled network compared against an ensemble of configuration models of the same network.

In the paper we examine the p-value distribution using our null model and the configuration model in 2-hop snowball sampled subnetworks. We repeated the comparison with all of the other sampling techniques using the method described in the main paper. The results can be seen in Figure 7. We use a χ^2 test to compare the distributions with the uniform distribution taking as observations the p-values of the statistic of interest from 1000 networks generated by selecting 25 random seeds (Table 3). We see that in all sampling techniques we reject the null hypothesis for the configuration model and for Snow1, Snow2 and all shortest paths we do not reject the null hypothesis for our null model. For Path3 in clustering we reject the null hypothesis at the 5% level but not at the 1% and further visual inspection of the distribution also does not draw any concern.

In the case of the clustering in Path2, triangles can occur only when two seed nodes are less than or equal to 2 hops apart and one of them is part of a triangle with $8,292$ nodes and an average shortest path length of 4.35 this is unlikely to happen. For non-continuous distributions, rather than the uniform distribution, we would expect to see the generalised inverse of the cumulative density function as distribution of the p-values. Fig. 8 shows that the distribution of average local clustering coefficients for Path2 networks sampled with 25 random seeds is indeed discontinuous and therefore we should not be surprised to see a non-uniform null distribution. In contrast with the same distribution from Snow1 shown in Fig. 9 is approximately continuously distributed and therefore the uniform distribution appears as the null distribution as expected.

While we cannot generalise from these results to all possible networks ensembles, and it is highly likely that there are network models and parameters ranges where the configuration model performs well in subnetworks, the configuration model does not perform well in general when comparing subnetworks based on seed lists. This demonstrates the need for an alternative to the configuration model for this task.

	Our Null		Configuration model	
	Assortativity	Clustering	Assortativity	Clustering
Path3	0.3115	0.0255	0	0
Path4	0.9659	0.0734	0	0
Shortest	0.8343	0.4788	0	0
Snow1	0.4654	0.4788	0	0
Snow2	0.2380	0.9522	0	0

Table 3: Goodness of fit χ^2 p-value results for different sampling techniques under our null model and the configuration model. 1000 networks are generated by selecting 25 random seeds and sampling with the given technique. Assortativity and average local clustering coefficient are calculated on the resultant network. We measure the p-value of these statistics under our null model and the configuration model. If the null hypothesis is correct and the distribution under the null hypothesis is continuous then these p-values, viewed as random observations, should be approximately uniformly distributed on the interval $[0, 1]$. This table gives the results from a χ^2 test for goodness to the uniform distribution. The results for the configuration model indicate that the distribution of p-values is far from uniform indicating that the configuration model is not a good null model , whereas in our model we do not reject the null hypothesis, lending support to its use as a null model.

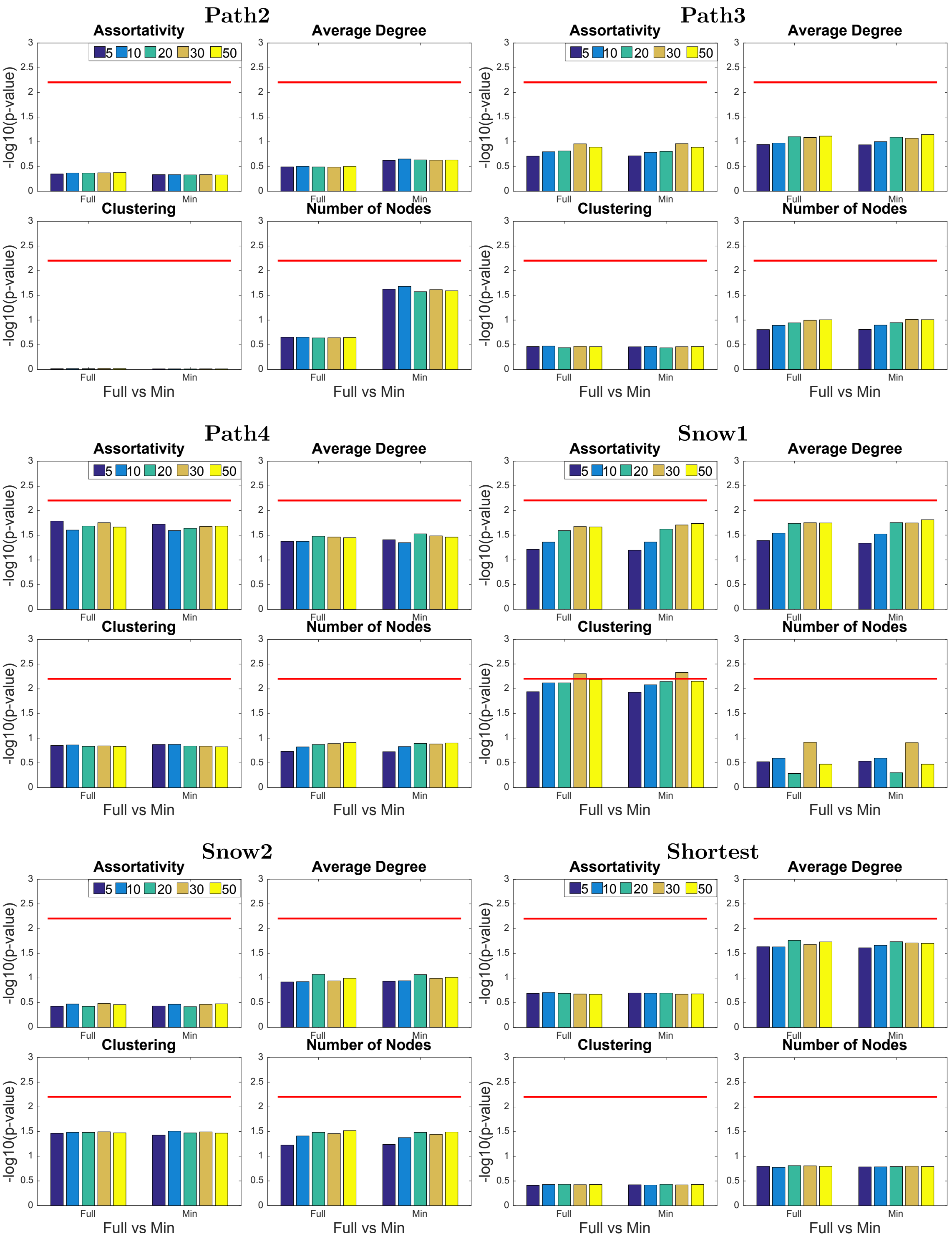

Figure 3: Test results for the OMIM seed list: smallest p-value, on a negative log scale under our null model. Multiple bars demonstrate the robustness of the method to the minimum number of items in each bin chosen when accounting for degree. Red: significance level (0.025/4). Left: Full seed list, right minimum seed list. Note negative logarithmic scale, e.g. only results that are above the Red significance line are considered significant. Only in Snowball 1 sampling is the result significant and only for the clustering coefficient, although this result is not robust to choice of bin size.

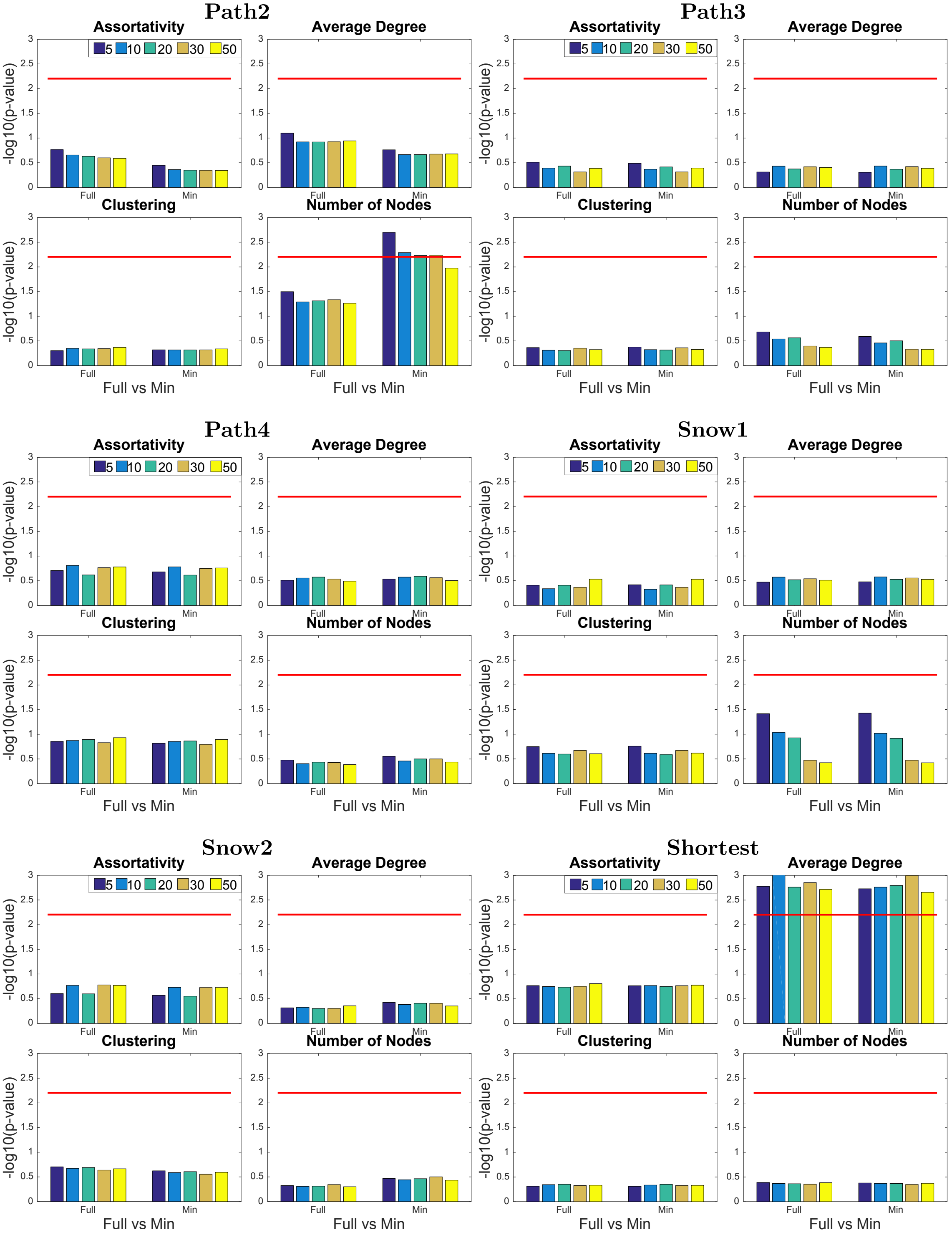

Figure 4: Test results for the Expression seed list: smallest p-value, on a negative log scale under our null model. Multiple bars demonstrate the robustness of the method to the minimum number of items in each bin chosen when accounting for degree. Red: significance level (0.025/4). Left: Full seed list, right minimum seed list. Note negative logarithmic scale, e.g. only results that are above the Red significance line are considered significant. Significant results are observed in number of nodes in Shortest path sampling and in most but not all bin sizes for the number of nodes in Path2 with the minimum seed list.

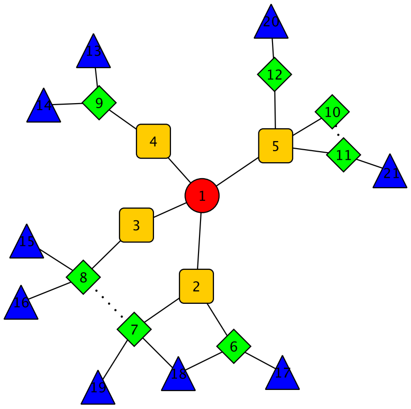

Figure 5: 2-Hop Snowball Sampling Example: The seed list consists of node 1 (circle), node shape represents distance from seed protein square, representing nodes 1 hop from the seed, diamond 2 hops from a seed, triangle 3 hops from a seed. Dashed edges represent cross-edges in a 2-hop snowball sample.

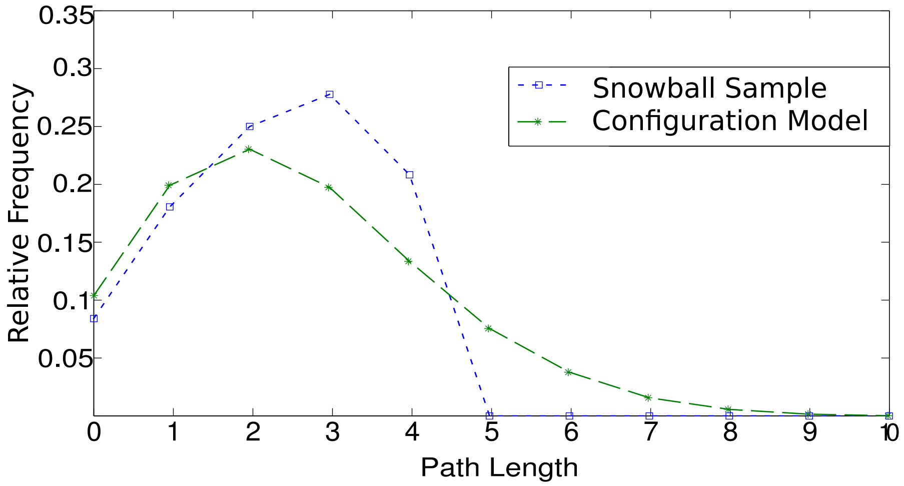

Figure 6: Comparison of distribution of shortest path lengths in the original network and over an ensemble of networks generated by a configuration model from the same network.

.

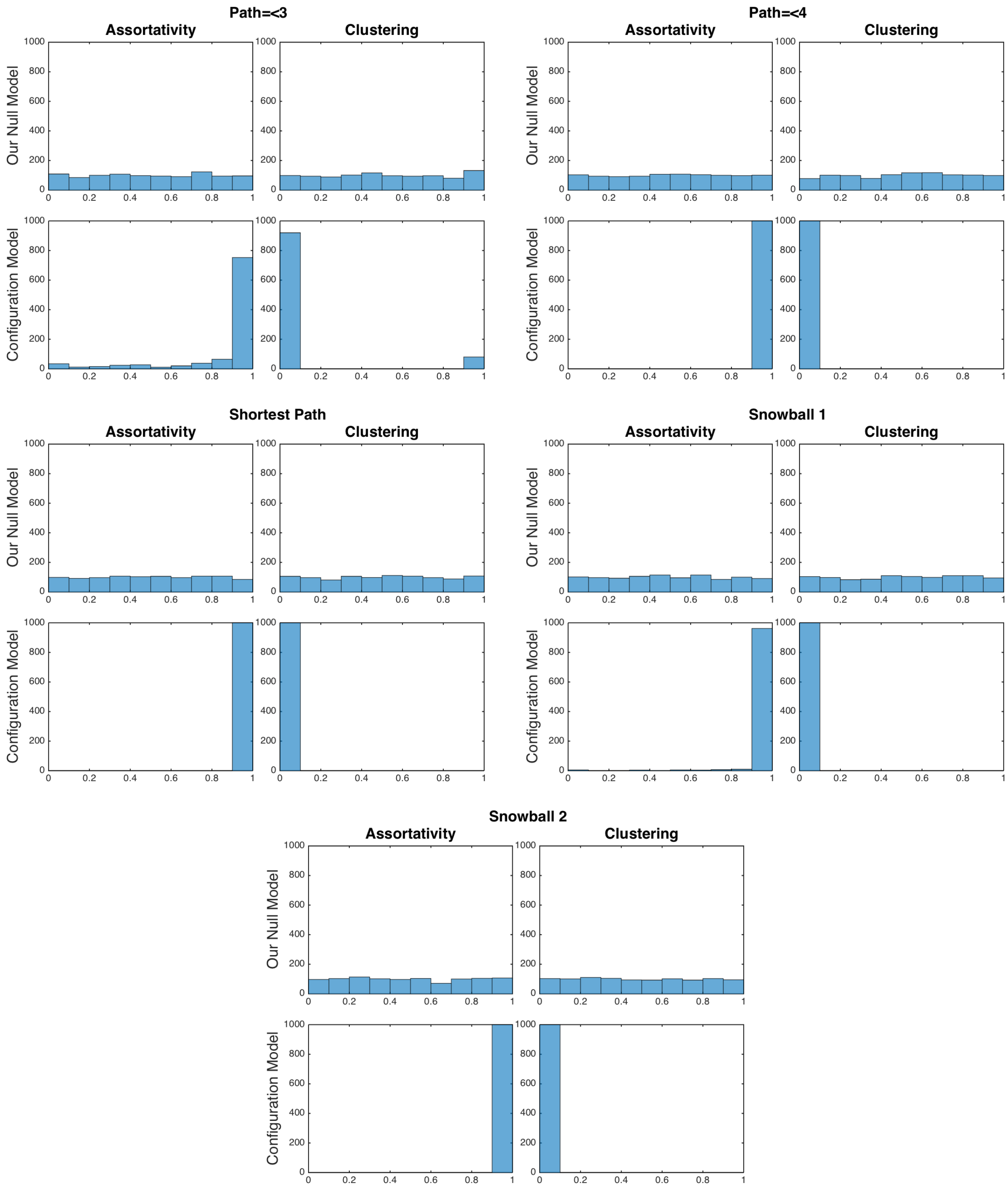

Figure 7: Distribution of p-value results for different sampling techniques under our null model and the Configuration Model. 1000 networks are generated by selecting 25 random seeds, assortativity and average local clustering coefficient are calculated. For each network we observe the p-value for deviation from a random network (our null model and the configuration model). For our null model the p-values are approximately uniformly distributed, indicating a reasonable fit of the null model. For the configuration model the distribution is far from uniform, indicating a poor fit.

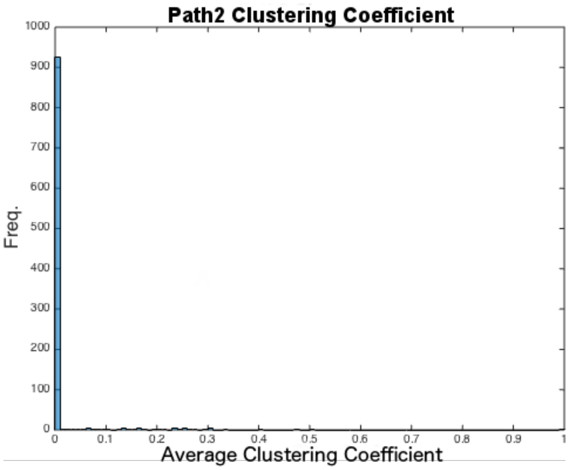

Figure 8: Distribution of Average Clustering Coefficient Path2 sampled from BioGRID network with 25 randomly chosen seeds. There is a strong concentration at 0 and a few discrete values which are not equal to 0.

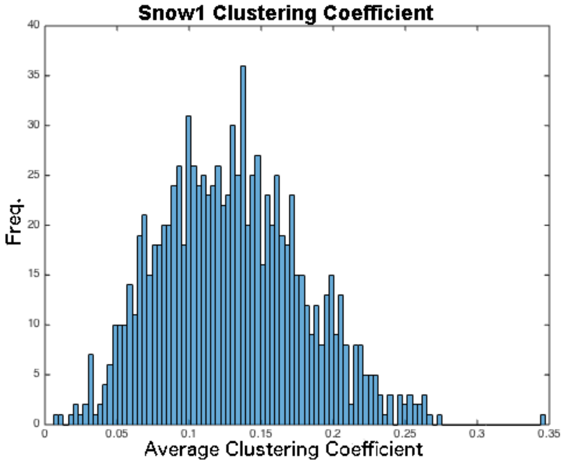

Figure 9: Distribution of Average Clustering Coefficient Snow1 sampled from BioGRID network with 25 randomly chosen seeds.

References

[1] Chatraryamontri,A. *et al.* (2013) The BioGRID interaction database: 2013 update. *Nucleic Acids Res*, **41** D816-D823.

[2] Conn,K.J. *et al.* (2003) cDNA microarray analysis of changes in gene expression associated with MPP+ toxicity in SH-SY5Y cells. *Neurochem Res*, **28** 1873–1881.

[3] Cormen,T.H. (2009) *Introduction to Algorithms* 3rd edn. MIT Press.

[4] Frank,O. (1977) Survey sampling in graphs. *J STAT PLAN INFER*, **1** 235–264.

[5] Hamosh,A. *et al.* (2005) Online mendelian inheritance in man (OMIM), a knowledgebase of human genes and genetic disorders. *Nucleic Acids Res*, **33 (suppl 1)** D514–D517.

[7] Stark,C. *et al.* (2006) BioGRID: a general repository for interaction datasets. *Nucleic Acids Res*, **34(suppl 1)** D535–D539.
